# Knowledge, attitudes, and beliefs regarding molar incisor hypomineralisation amongst Swiss dental students

**DOI:** 10.1186/s12903-021-01911-7

**Published:** 2021-10-26

**Authors:** Blend Hamza, Karim Elhennawy, Hubertus van Waes, Spyridon N. Papageorgiou

**Affiliations:** 1 Clinic of Orthodontics and Pediatric Dentistry, Center of Dental Medicine, University of Zurich, Plattenstrasse 11, 8032 Zurich, Switzerland; 2 Department of Orthodontics, Dentofacial Orthopedics and Pedodontics, Charité – Universitätsmedizin Berlin, Corporate Member of Freie Universität Berlin, Humboldt-Universität, and Berlin Institute of Health, Aßmannshauser Str. 4-6, 14197 Berlin, Germany

**Keywords:** Molar incisor hypomineralisation, Perception, Awareness, Dental students

## Abstract

**Background:**

Knowledge obtained at the undergraduate level regarding molar incisor hypomineralisation (MIH) has an impact on future practice of dentists and paediatric dentists. This cross-sectional study aimed to assess final-year dental students’ knowledge, attitudes and beliefs towards MIH in all Swiss universities.

**Methods:**

A previously utilised survey (in both English and German) was distributed among final-year dental students in all Swiss dental schools (Basel, Bern, Geneva and Zurich). It probed students’ knowledge, attitudes and beliefs regarding the diagnosis, prevalence, aetiology, and management of MIH, and was structured in two parts: knowledge/perception and clinical application. The students’ responses were analysed statistically with descriptive statistics.

**Results:**

113 out of 133 final-year Swiss dental students took part in the study (85%). Nearly all students were familiar with MIH (99%), but only 12% of them felt confident when diagnosing MIH clinically. Direct composite fillings (66%), indirect restorations (28%) and preformed stainless-steel crowns (26%) were chosen as most suitable treatment options for MIH-affected teeth.

**Conclusion:**

Final-year Swiss dental students are well informed about MIH. However, they report low level of confidence when clinically confronted with MIH-affected teeth regarding its diagnosis and treatment. Swiss Universities curricula should be revisited accordingly.

## Background

The term Molar-Incisor Hypomineralisation (MIH) was first suggested in 2001 by Weerheijm et al., to describe demarcated enamel lesions mainly encountered in the first permanent molars with frequent involvement of upper permanent incisors [1]. MIH-affected enamel has reduced mineral density, higher protein content, and shows more porosity in comparison to sound enamel [2]. Furthermore, etch patterns of MIH-affected enamel are less retentive than those of sound enamel, leading to higher failure rates at the enamel-adhesive interface and loss of the filling [3]. All aforementioned issues, amongst others, make MIH-affected teeth a challenging condition for patients and dentists [4].

A handful of factors have been hypothesised to be at least partly responsible for the aetiology of MIH. These include, but are not limited to: genetic factors, exposure to environmental pollutants, common childhood illnesses, and birth complications [5]. The prevalence of MIH also shows a wide variation ranging between 2 and 40% [6], with an estimated pooled prevalence of MIH at 14.2% globally [7], and which indicates that most dentists will at some time during their career encounter MIH-affected teeth.

It is safe to assume that the knowledge and experience acquired during the undergraduate level shapes the perception and behaviour of future dentists in dealing with clinical challenges.

As MIH continues to pose a serious clinical challenge for both patients and dentists [8–10], it is necessary to probe the perception and knowledge of future dentists regarding its diagnosis and management. This study was therefore carried out to investigate the knowledge, attitudes, and beliefs of final-year Swiss dental students towards MIH.

## Material and methods

The current study is reported in accordance with the Strengthening the Reporting of Observational Studies in Epidemiology (STROBE) statement [11]. A previous survey, which was already validated in both German and English [12] was distributed to final-year dental students in Switzerland to assess their knowledge, attitudes and beliefs regarding MIH. Ethical approval for this anonymised optional questionnaire survey was obtained from Zurich cantonal ethics committee (BASEC-Nr. Req-2021-00792). Participants consented to participate in the study and for the collected data to be published by returning the completed survey. A nationwide census sample of final-year students from all four dental schools in Switzerland (Basel, Bern, Geneva and Zurich) was established (n = 133). The head or a senior researcher in the department of paediatric dentistry in each university was contacted by the first author via email and invited to take part in the study. Students in the University of Geneva received the English version of the survey, while other universities received the German version. Surveys were administered and data were collected between February and June 2021.

The survey started with a brief description of MIH with clinical photos, followed by demographic questions about the participants and then by specific questions on MIH regarding its diagnosis, prevalence, students’ attitude and beliefs towards its management and educational need. A native German speaker translated the original English survey into German, which was then back translated into English by an independent English native speaker. To estimate the intra-rater reliability (κ = 0.78), the final survey was piloted among 3^rd^-year dental students (n = 40, Charité, Berlin) who were asked to re-take the survey after one month.

### Statistical analysis

Following data extraction by a single author, data was analysed statistically with descriptive statistics, after checking the distribution of continuous outcomes graphically and with the Shapiro–Wilk test. Categorical variables were summarised with absolute/relative frequencies, while non-normally distributed continuous variables were summarised with medians and their Interquartile Range (IQR). All analyses were done in StataSE 13.0 (StataCorp, College Station, TX, USA) and the anonymised dataset was openly available through Zenodo (10.5281/zenodo.5159386).

## Results

All invited dental schools in Switzerland agreed to participate in the study. In total, 113/133 final-year dental students (85% of the total number of final-year dental students in Switzerland in the academic year 2020/2021) answered and returned the survey. As this was an optional and anonymous survey, the students who did not participate (n = 20) and their reasons remain unclear. Participants were on average 25-year-old (range 22 to 36 years; IQR 24–26 years) and 61% of them (n = 69) were female. The geographic distribution of the participants according to their universities was as follows: Basel (22%; n = 25), Bern (36%; n = 40), Geneva (14%; n = 16) and Zurich (28%; n = 32).

Table 1 summarises the responders’ answers regarding the various aspects of MIH. Almost all Swiss dental students (99%) had already heard of MIH. This knowledge was mostly collected from lectures (92%) or during clinical courses (50%). Although the majority of the students (98%) were familiar with the clinical features of MIH, only 35% of them reported themselves able to diagnose a patient with MIH clinically. Moreover, 88% of the students were either only slightly confident (46%) or not confident at all (42%) when diagnosing MIH clinically; 72% had difficulties distinguishing MIH from other dental defects, especially amelogenesis imperfecta and enamel hypoplasia. Ninety percent of the students estimated the prevalence of MIH in Switzerland to lay between 0 and 25%, but only 20% of them reported knowing this exact percentage as a fact. Most of the students (95%) reported having seen MIH-affected teeth in less than 10% of their patients.

**Table 1:** Students’ responses to the survey questions

Question	Total response rate (%)	Percentage distribution of positive answers: n (%)
1. Are you familiar with MIH?	100	Yes: 112 (99%)/No: 1 (1%)
1″. If so, how did you hear about it?^†^	100	Dental journal: 15 (13%) Lecture: 104 (92%) Lecture notes: 40 (35%) Brochures: 2 (2%) Internet: 19 (17%) Books: 14 (12%) Clinical courses: 56 (50%) Other students: 12 (11%) Other: parents; paediatric dentist; radio
Do you know the clinical features of MIH?	100	Yes: 111 (98%)/No: 2 (2%)
3. Do you have any difficulty distinguishing MIH from other tooth malformations?	99	Yes: 81 (72%)/No: 31 (28%)
3″. If so, which?^†^	99	Dental fluorosis: 26 (23%) Enamel hypoplasia: 55 (49%) Amelogenesis imperfecta: 65 (58%) Dentinogenesis imperfecta: 25 (22%)
4. Which factors do you think are involved in the aetiology of MIH?^†^	99	Genetic factors: 92 (82%) Chronic medical condition(s) that affect the mother during pregnancy: 52 (46%) Chronic medical condition(s) that affect the child: 41 (37%) Antibiotics/medications taken by the mother during pregnancy: 42 (38%) Antibiotics/medications taken by the involved child: 23 (21%) Environmental contaminants: 48 (43%) Acute medical condition(s) that affect the mother during pregnancy: 37 (33%) Acute medical condition(s) that affect the involved child: 28 (25%) Fluoride exposure: 5 (4%) None: 1 (1%) Other: No one knows; defect of mineral content; unclear; plasticisers
5. Do you know the prevalence of MIH in Switzerland?	98	Yes: 22 (20%)/No: 89 (80%)
5″. Where do you estimate the prevalence of MIH in Switzerland to be?	97	0–25%: 99 (90%)/25–50%: 11 (10%)/> 50%: 0 (0%)
6. Do you think it is worth investigating the MIH prevalence in Switzerland?	100	Yes: 103 (91%)/No: 10 (9%)
7. Are you capable of diagnosing a patient with MIH?	99	Yes: 39 (35%) No: 6 (5%) Not sure: 67 (59%)
8. How often do you see teeth with MIH in your clinical courses?	100	Never: 56 (50%) Weekly: 2 (2%) Monthly: 11 (10%) Yearly: 44 (39%)
9. In what proportion of your patients do you observe MIH teeth approximately?	97	< 10%: 104 (95%) 10–25%: 5 (5%) > 25%: 1 (1%)
10. Which of the following features do you see most frequently in MIH teeth?	98	White defects: 27 (24%) Yellow–brown defects: 63 (57%) Defects with enamel loss: 8 (7%) Other: combination of above (× 9); never seen (× 4)
11. How confident do you feel when diagnosing MIH?	100	Very confident: 0 (0%) Confident: 14 (12%) Slightly confident: 52 (46%) Not confident at all: 47 (42%)
12. Do you know if there are clinical criteria to diagnose MIH?	99	Yes, and I know how to use clinically: 34 (30%) Yes, but I don’t know how to use clinically: 56 (50%) No: 22 (20%)
13. Have you seen demarcated hypomineralised permanent teeth defects in your patients that were not the first permanent molars and incisors?	99	Yes: 29 (26%)/No: 83 (74%)
13″. If so, please name the tooth		Premolars (× 10); canines (× 7); 2^nd^ molar (× 4); deciduous teeth (× 2); all teeth
14. How often have you noticed demarcated hypomineralised lesions in the second primary molars in comparison to the first permanent molar tooth?	100	More often: 4 (4%) Less often: 28 (25%) As common as the 1st permanent molar: 5 (4%) Never seen: 76 (67%)
15. Which material do you think is best for treating MIH molars?^†^	100	Amalgam: 1 (1%) Composite: 75 (66%) Flowable composite: 33 (29%) Resin-modified glass ionomer cement: 19 (17%) Compomer: 6 (5%) Glass ionomer cement: 25 (22%) Preformed stainless-steel crown: 29 (26%) Indirect restoration (CEREC): 32 (28%) Other: Ceramic; crown (2x); depends on severity; fluoride; Fuji Triage (2x); Icon; orthodontic-bands
16. Which factors influence your choice of restorative material?^†^	100	Adhesion: 90 (80%) Aesthetics: 57 (50%) Patient/parent preference: 33 (29%) Durability: 70 (62%) Remineralising potential: 39 (35%) Hypersensitivity: 46 (41%) Personal experience: 22 (19%) Research findings: 42 (37%)
17. Do you think MIH is a relevant clinical problem?	100	Yes: 107 (95%)/No: 6 (5%)
17″. If so, what are your difficulties?^†^	100	Diagnosis: 66 (58%) Aesthetics: 32 (28%) Achieving adequate local anaesthesia: 14 (12%) Determining the restoration margins of affected enamel: 27 (24%) Providing adequate restoration: 49 (43%) Long-term restoration success: 69 (61%) Achieving patient comfort (for function and oral hygiene: 43 (38%)
18. Would you suggest including clinical training regarding MIH in your clinical course?	100	Yes: 101 (89%)/No: 12 (11%)
18″. In which area(s) do you think you need to know/be taught about the most?^†^	100	Diagnosis: 90 (80%) Aetiology: 46 (41%) Treatment: 97 (87%) Other: long-term prognosis; practical treatment; prevention

^†^Multiple choices were allowed

The most common MIH feature seen by the students were yellow/brown demarcations, followed by white demarcations and post-eruptive enamel breakdown. Only one-quarter of the students (26%) had encountered demarcated hypomineralised defects in permanent teeth other than the first permanent molars and incisors, mainly in premolars. More than two-thirds of the students (67%) had never seen MIH-similar lesions in primary second molars. The majority of the students (82%) held genetic factors responsible for the aetiology of MIH, followed by chronic diseases affecting the mother during pregnancy (46%), environmental contaminants (43%) and antibiotics/medications taken by the mother during pregnancy (38%).

Regarding treatment options of MIH-affected teeth, 66% of the students believed direct resin composite to be a suitable treatment option, followed by indirect Computer-Aided Design and Computer-Aided Manufacturing (CAD/CAM) restorations (28%), and preformed stainless-steel crowns (26%). The main factors that influenced the students’ choice of treatment materials were adhesion (80%), durability (62%), and aesthetics (50%).

The vast majority of students (95%) believed that MIH was a serious clinical problem, mostly regarding the possibility of achieving a long-term restoration success (61%) and diagnosis (58%). Eighty-nine percent of the students suggested including more clinical training on MIH-affected teeth in the curriculum, especially regarding treatment (87%), diagnosis (80%) and aetiology (41%).

## Discussion

This is, to our knowledge, the first nationwide study to report on the knowledge, attitudes, and beliefs of final-year dental students toward MIH in Switzerland. The basic idea behind such studies is to report any knowledge gaps that need to be addressed during the undergraduate studies, where the fundamental competence and the know-how of future dentists is shaped. This study shows that Swiss final-year students are theoretically familiar with MIH, but lack the confidence when it comes to clinically diagnose and manage it. A statistical analysis to point out the differences between the four Swiss dental schools was deemed unnecessary. However, and for what it is worth, the answers of the students from all dental schools did not seem to be systematically different.

The survey used in this study has been pre-validated and distributed among dental students and/or dentists in earlier studies [13–16].As the dental school in Geneva was the only non-German speaking school in Switzerland, the students there received the English version of the survey. The authors were assured that the 26 final-year dental students in Geneva had a good English command, and thus, translating the survey into French was not considered necessary. Eighty-five percent of final-year dental students in all Swiss universities answered and returned the survey. This relatively high participation rate makes the results derived from the study robust and representative of the Swiss population. Moreover, the students took part in the study during the last couple of months before their graduation, which gives a realistic insight into the knowledge and perception they will clinically implement as young dentists. On the other hand, it could be speculated that students chose answers they deemed “desirable” or “right” rather than those, which really reflect their knowledge and attitude. This factor is almost always present in survey-studies, even though the anonymous and optional nature of the survey could have helped reduce its effect.

Ninety-nine percent of Swiss students had heard of MIH during their undergraduate study. A similar percentage of students also reported themselves to be familiar with MIH in Germany (99%) [12]. In Saudi Arabia, a much lower percentage of final-year dental students reported themselves being familiar with MIH (28.4%) [16]. Nevertheless, the Saudi study was carried out only in one dental school, and thus might not represent the actual average knowledge status for the entire country, but rather be associated with the structure of a specific university curriculum. In general, Swiss and German final-year students reported similar knowledge regarding the diagnosis of MIH. Eighty-eight percent of Swiss students and 85% of German students reported themselves not to be confident when diagnosing MIH clinically. Amelogenesis imperfecta and enamel hypoplasia were reported to be the malformation most difficult to be distinguished from MIH-affected teeth in both countries. This high level of uncertainty of MIH diagnosis explains the high percentage of Swiss and German students (ca. 90%) who claim to include additional clinical training on MIH in their curriculum.

Most Swiss students predicted the prevalence of MIH in Switzerland to be between 0 and 25%, which is in accordance with the globally estimated pooled prevalence of MIH (13.1–14.2%) [7, 10]. Two thirds of Swiss students never saw MIH-affected primary molars. The same percentage was also reported by German students [12]. Genetic factors were considered the most involved in the aetiology of MIH in the present study, which is similar to the results of both German and the Saudi Arabian studies [12, 16].

Regarding the clinical management of MIH-affected teeth, direct composite filling was chosen as the most suitable treatment choice by most Swiss and German students (66% and 70%, respectively). Preformed stainless-steel crowns were the fourth most preferable treatment choice in this study (26%), whereas more German students chose it as a treatment option (39%, second most preferable choice). Differences in the preferred treatment options for MIH-affected teeth might also be associated with the differential insurance coverage of some dental procedures between the two countries.

It is safe to assume—based on the present results—that undergraduate students in Switzerland need more clinical training regarding the clinical diagnosis and management of MIH. It could be interesting to distribute the same survey used in this study among well-experienced dentists to figure if post-graduate studies, continuous-education courses or even learn-by-doing in the praxis bring any further knowledge regarding MIH. Similar nationwide surveys in other parts of the globe could also shed light on different management approaches regarding MIH-affected teeth. This might initiate a beneficial academic exchange between dental schools. It could be argued that some questions in the survey could be modified in future surveys to obtain more specific answers. However, going into details in every aspect/question, might result in very long and tiring-to-fill surveys and significantly affect the number of participants.

## Conclusion

Based on the results of this study, knowledge, attitudes and beliefs of Swiss final-year dental students are in need of improvement. Swiss Universities’ curricula should be revisited to include more educational material about the diagnosis/management of MIH-affected teeth in order to increase the confidence of future dentists when confronted with such challenge.

## Data Availability

The datasets used and/or analysed during the current study are available from the corresponding author on reasonable request.

## Abbreviation

MIH: Molar Incisor Hypomineralisation
